# Aerosol Exposure to Rift Valley Fever Virus Causes Earlier and More Severe Neuropathology in the Murine Model, which Has Important Implications for Therapeutic Development

**DOI:** 10.1371/journal.pntd.0002156

**Published:** 2013-04-04

**Authors:** Christopher Reed, Kenny Lin, Catherine Wilhelmsen, Brian Friedrich, Aysegul Nalca, Ashley Keeney, Ginger Donnelly, Joshua Shamblin, Lisa E. Hensley, Gene Olinger, Darci R. Smith

**Affiliations:** United States Army Medical Research Institute of Infectious Diseases (USAMRIID), Fort Detrick, Maryland, United States of America; University of North Carolina at Chapel Hill, United States of America

## Abstract

Rift Valley fever virus (RVFV) is an important mosquito-borne veterinary and human pathogen that can cause severe disease including acute-onset hepatitis, delayed-onset encephalitis, retinitis and blindness, or a hemorrhagic syndrome. Currently, no licensed vaccine or therapeutics exist to treat this potentially deadly disease. Detailed studies describing the pathogenesis of RVFV following aerosol exposure have not been completed and candidate therapeutics have not been evaluated following an aerosol exposure. These studies are important because while mosquito transmission is the primary means for human infection, it can also be transmitted by aerosol or through mucosal contact. Therefore, we directly compared the pathogenesis of RVFV following aerosol exposure to a subcutaneous (SC) exposure in the murine model by analyzing survival, clinical observations, blood chemistry, hematology, immunohistochemistry, and virus titration of tissues. Additionally, we evaluated the effectiveness of the nucleoside analog ribavirin administered prophylactically to treat mice exposed by aerosol and SC. The route of exposure did not significantly affect the survival, chemistry or hematology results of the mice. Acute hepatitis occurred despite the route of exposure. However, the development of neuropathology occurred much earlier and was more severe in mice exposed by aerosol compared to SC exposed mice. Mice treated with ribavirin and exposed SC were partially protected, whereas treated mice exposed by aerosol were not protected. Early and aggressive viral invasion of brain tissues following aerosol exposure likely played an important role in ribavirin's failure to prevent mortality among these animals. Our results highlight the need for more candidate antivirals to treat RVFV infection, especially in the case of a potential aerosol exposure. Additionally, our study provides an account of the key pathogenetic differences in RVF disease following two potential exposure routes and provides important insights into the development and evaluation of potential vaccines and therapeutics to treat RVFV infection.

## Author Summary

Rift Valley fever (RVF) is an important neglected tropical disease that has caused severe epidemics and epizootics throughout Africa and the Arabian Peninsula. Severe outbreaks have involved tens of thousands of both human and livestock cases for which no effective, commercially available human vaccines or antiviral drugs are available. Mosquito transmission is the primary means for human infection although it can also be transmitted by aerosol or through mucosal contact. In this study, we directly compared the pathogenesis of RVF virus (RVFV) following aerosol exposure to a peripheral exposure in the mouse model. Additionally, we evaluated the effectiveness of ribavirin, a known antiviral compound, administered prior to virus exposure. The virus affected the liver despite the route of exposure. However, viral invasion of brain tissues occurred much earlier and was more severe in mice exposed by aerosol compared to peripherally exposed mice. Mice exposed peripherally and treated with ribavirin were partially protected whereas treated mice exposed by aerosol were not protected. Our results have important implications for understanding the pathogenesis of RVFV and for the development and evaluation of potential vaccines and therapeutics.

## Introduction

Rift Valley fever virus (RVFV) is a negative sense, single-stranded RNA virus of the *Bunyaviridae* family, genus *Phlebovirus*. An important pathogen of humans and domesticated animals, the virus may be transmitted when an infected mosquito feeds on a host, by contact with tissues, blood, or fluids from infected animals, or by exposure to infectious aerosols, such as those generated in abattoirs during handling of infected animals [1]. RVFV was first isolated during a 1930 epizootic in East Africa [2]. Subsequent outbreaks ranged from South Africa to Egypt, and west to Senegal [3]. Until the 21^st^ century, it was assumed that RVFV was confined primarily to continental Africa; however, epidemics in Saudi Arabia and Yemen after unusually heavy rainfall in 2000 indicate the possibility of a RVFV introduction occurring wherever a competent vector exists [4]. Additionally, productive experimental infection of mosquitoes from multiple distinct geographical regions (including the most widespread vector, *Culex pipiens*) reinforces the feasibility of accidental or intentional import of RVFV from endemic regions with subsequent maintenance in nascent vector and host populations [5]–[8].

In livestock the susceptibility and virulence differ by host species and age [3]. Spontaneous abortion is such a common consequence of infection among domesticated animals that the resulting abortion “storms” arising during epizootics are often the first recognized sign of a RVFV outbreak [9]. Necrotizing hepatitis, encephalitis, and death commonly occur in infected livestock. The disease in humans is generally a self-limiting, mild influenza-like condition with fever. However, a smaller but still poorly defined percentage of individuals develop a severe illness characterized by acute-onset hepatitis, delayed-onset encephalitis, retinitis, blindness, or a hemorrhagic syndrome [3]. While it has been traditionally reported that 1%–2% of human infections result in severe disease, case-fatality rates have varied between epidemics and are generally estimated to range from 10–20% for hospitalized individuals [10]–[12].

In conjunction with its infectiousness and pathogenicity in humans, the range and prolificacy of its host species highlight the importance of RVFV as a potential agent of bioterrorism. Furthermore, there are currently no licensed vaccines or approved antivirals for preventing or treating RVF in humans, and its infectiousness by aerosol exposure coupled with ease of proliferation adds to the threat posed by intentional dissemination of RVFV. As such, RVFV is listed as a select agent by the Centers for Disease Control and Prevention (CDC), and is classified as a Department of Health and Human Services (HHS) and United States Department of Agriculture (USDA) overlap select agent.

Several animal models of RVFV infection have been described [13]. Recently, we completed a detailed study to characterize the pathogenesis of RVFV in the BALB/c mouse model [14], [15], which showed important similarities to severe human infections. Infection of BALB/c mice with RVFV subcutaneously (SC) resulted in high-titer viremia and demonstrated RVFV tropism for a variety of tissue and individual cell types on the basis of histopathology, immunohistochemistry, and electron microscopy. A major consequence of infection was overwhelming infection of hepatocytes that subsequently underwent apoptosis. Most mice succumbed to RVFV between days 3 and 6 post-infection (PI) which we attributed primarily to severe hepatitis as indicated by the overwhelming infection of hepatocytes and increase in high levels of hepatic enzymes in the blood. The remaining mice were able to effectively clear virus from the liver and blood, but exhibited neuroinvasion and developed lethal panencephalitis [14].

The route of neuroinvasion in our mouse model was unclear and we postulated one potential route may have occurred by way of the olfactory nerves leading to infection of the olfactory bulbs, which was based on our observation that olfactory neurons lining the nasal tract are a target of RVFV. We therefore hypothesized that the incidence and severity of central nervous system infection subsequent to aerosol infection may be significantly higher than is seen with a peripheral (SC) infection. RVFV is transmitted easily by aerosol to humans, which is evident by the number of laboratory workers who have become infected [16] and the potential for infection of veterinarians and abattoir workers who handle infected animals. Mice are known to be highly susceptible to RVFV aerosol exposure [17]. However, detailed studies describing the pathogenesis of RVFV after aerosol exposure have never been completed. Additionally, potential antivirals have not been evaluated for effectiveness following an aerosol exposure to RVFV.

Due to the possibility of infection by aerosol exposure (such as during a bioterrorism event), we have expanded our studies of pathogenesis to include mice exposed to a lethal dose of virus by aerosolization. Additionally, we evaluated the effectiveness of ribavirin, an antiviral compound with known activity against RVFV both *in vitro* and *in vivo* [18]–[21]. By comparing the course of disease after aerosol and SC RVFV exposures in the presence and absence of antiviral treatment, we determined the suitability of the mouse model in antiviral therapeutics trials and provided further insight into the efficacy of ribavirin to treat RVFV infection.

## Materials and Methods

### Ethics Statement

Research was conducted under an IACUC approved protocol in compliance with the Animal Welfare Act, PHS Policy, and other Federal statutes and regulations relating to animals and experiments involving animals. The facility where this research was conducted is accredited by the Association for Assessment and Accreditation of Laboratory Animal Care, International and adheres to principles stated in the Guide for the Care and Use of Laboratory Animals, National Research Council, 2011.

### Animals

Female BALB/c mice were obtained from the National Cancer Institute, Frederick Cancer Research and Development Center (Frederick, MD), and were used at 6–8 weeks old. Mice were housed in microisolator cages and were provided water and chow *ad libitum*.

### Viral Strain

Recombinant viral strain ZH501 was rescued as previously described [22], and the exact complete genome sequence was confirmed by techniques described previously by Bird et al. [23]. Strain ZH501 was originally isolated from a fatal human case during the 1977 epidemic in Egypt. In this study, the lethal dose at 50% (LD_50_) of this strain was determined in BALB/c mice infected SC or by aerosol. To determine the LD_50_, five cohorts of mice comprised of 10 mice each were infected with a range of doses (1000-0.1 PFU) and monitored for survival.

### Aerosol Exposure

Prior to animal exposure studies, a sham aerosol spray using only the virus was performed in order to calculate a spray factor. The resulting spray factor was used to calculate the starting concentration of the virus necessary to obtain the target dose. Groups of ten unanesthetized mice were exposed to aerosolized RVFV created by a three-jet collision nebulizer (BGI Inc., Waltham, MA) for 10 min at a constant flow rate of 19 L/min in a whole-body exposure chamber housed within Class III biological safety cabinets in a biosafety level-3 suite by using automated aerosol exposure system. The particle sizes generated with this system has an average of 1–2 µm mass median aerodynamic diameter. Relative humidity was a steady state of 43–65% and temperature was ambient (approximately 20–22°C). Actual exposures received by each group of animals were determined by performing standard plaque assays on samples collected from an all-glass impinger (AGI; Ace Glass, Vineland, NJ). Complete medium with antifoam A 0.001% w/v (Sigma, St. Louis, MO) was used as collection medium in the impinger for titration by standard plaque assay. The dose was calculated using the following formulas: Dose = [Aerosol] (µg/mL)×minute volume (mL)×exposure time (min); minute volume = 2.1(weight (g))^0.75^.

### Pathogenesis Study Design

Mice were exposed to a target dose of 1000 PFU of RVFV either by aerosolization as described above or by SC injection in a total volume of 100 µL. Two cohorts of mice were used for the pathogenesis study design. One cohort of mice (n = 100) were used to monitor survival and collect blood/tissue samples when the mice exhibited terminal (end-stage) signs of disease (these mice received an actual dose of 1600 PFU). The second cohort of mice (n = 200) was used to serially sample mice (randomly selected) on days 1–8 PI (these mice received an actual dose of 646 PFU). All mice were implanted with IPTT-300 temperature chips (Biomedic Data Systems, Seaford, DE) to identify individual animals and monitor body temperature throughout the studies. Temperature and signs of clinical disease were observed daily, and one group of mice from both the SC and aerosol exposure groups (n = 4/day PI) was selected for retroorbital blood sampling to analyze viremia, blood chemistry, and hematology. These mice were then perfused with PBS (to remove virus contaminated blood from tissues) and tissues were collected for virus titration by standard plaque assay and histology. Tissues collected for titration were weighed and homogenized in Eagle's minimal essential medium (EMEM) containing 5% fetal bovine serum and gentamicin. Tissues were homogenized using the Qiagen Tissue Lyser II (Qiagen, Valencia, CA) followed by centrifugation at 9,000× g for 10 min and the supernatant stored at −70°C until further evaluation by standard plaque assay to determine titers.

### Evaluation of Antiviral Activity of Ribavirin Following Aerosol and SC RVFV Exposures

Mice were treated by intraperitoneal (IP) injection with PBS (n = 30; drug delivery vehicle control) or with ribavirin (n = 30; Sigma; 100 mg/kg) beginning two hours prior to virus exposure and continuing once a day thereafter for 9 days (10 days total). This dose was selected based on a previous study which determined that 100 mg/kg of ribavirin administered prophylactically was protective in mice and higher doses of ribavirin were found to be toxic [21]. Mice were infected either by aerosol (n = 10/treatment group) or SC exposure (n = 10/treatment group) with a target dose of 1000 PFU (actual dose was 1951 PFU) or remained as uninfected controls (n = 10/treatment group). All mice were monitored for changes in weight, temperature, and survival.

An additional cohort of mice was treated with PBS or ribavirin and infected either by aerosol (n = 15/treatment group) or SC (same as described above; n = 15/treatment group) for a comparative pathology study on days 3, 5, and 8 PI. These time-points were chosen based on previous pathogenesis studies that indicate that these are key days for RVFV infection in this model; and that they reflect early, middle, and late disease states [14]. Mice were bled retroorbitally for virology, hematology, and blood chemistry analyses (n = 5/day PI). Immediately following blood sampling and under deep anesthesia, mice were perfused with PBS to remove residual blood in tissues. Tissues were then collected for titration by standard plaque assay as described above.

### Plaque Assays

Plaque assays for RVFV used 90–100% confluent Vero cells (American Type Culture Collection, Manassas, VA) in 12-well plates. Samples for titration were serially diluted 10-fold in culture medium, and then 100 µL of each dilution was added to each of two wells. Plates were rocked every 15 minutes during 1 hour of incubation at 37°C with 80% relative humidity and 5% CO_2_. After incubation, a primary overlay of 0.5% agarose and 5% fetal bovine serum in 1× Earle's minimum essential medium (EMEM) was added to each well. Plates were then incubated for an additional 3 days before addition of a secondary overlay, which contained 4% neutral red in addition to the formula of the primary overlay. After addition of neutral red stain, plates were incubated for an additional 24 hours, and plaques were counted 4 days PI.

### Hematology and Blood Chemistries

Whole blood was added either to an EDTA tube (Safe-T-Fill, RAM Scientific Inc., Yonkers, NY) for complete blood count (CBC) determination using a Hemavet (Drew Scientific, Dallas, TX) or whole blood was added to a lithium heparin tube (Safe-T-Fill) for clinical chemistry analysis using the comprehensive diagnostic panel analyzed on a Vetscan (Abaxis, Union City, CA). The Hemavet measured the following parameters: white blood cells (WBC), total number and percent of neutrophils, lymphocytes, macrophages, eosinophils, and basophils, red blood cells (RBC), hemoglobin (Hb), hematocrit (HCT), mean corpuscular volume (MCV), mean corpuscular hemoglobin (MCH), mean corpuscular hemoglobin concentration (MCHC), red cell distribution width (RDW), platelets (PLT), and mean platelet volume (PLT). The vetscan determined levels of alanine aminotransferase (ALT), albumin (ALB), alkaline phosphatase (ALP), amylase (AMY), total calcium (CA^++^), creatinine (CRE), globulin (GLOB), glucose (GLU), phosphorus (PHOS), potassium (K^+^), sodium (NA^+^), total bilirubin (TBIL), total protein (TP), and urea nitrogen (BUN).

### Histopathology

Mice were euthanized by perfusion under deep anesthesia and a complete necropsy was performed to collect a full complement of tissues for histopathology. Tissues were fixed in 10% neutral-buffered formalin for a minimum of 21 days, and then removed from biocontainment and processed for routine histopathology. Paraffin-embedded tissues were sectioned and then stained with hematoxylin and eosin (HE). Duplicate tissue sections were stained by immunohistochemistry for RVFV antigen using a kit (EnVision System, Dako Corp., Carpinteria, CA). Sections were deparaffinized and pretreated with proteinase K for 6 minutes before incubation with primary anti-RVFV antibody (R1547, rabbit polyclonal), diluted 1∶800 and incubated at room temperature for 30 minutes. This was followed by incubation with a peroxidase-conjugated, polymer-based secondary antibody. Tissues stained by IHC were examined microscopically to determine the types of cells labeled for viral antigen.

### Statistical Analysis

SAS version 9.1.3 (SAS Institute Inc., Cary, NC) was used to determine differences in the mean blood chemistry and hematology results between day −1 or 0 (uninfected controls) and subsequent time points using T-tests with step-down bootstrap adjustment. Signed rank tests with step-down Sidak adjustment was used for comparisons of temperature and weight from day 0 to subsequent time points within each study and Wilcoxon-Mann-Whitney tests for comparisons of temperature and weights between exposure routes (aerosol and SC) at each time point. A two-way ANOVA was used to compare viral titers between exposure groups.

## Results

### Survival and Clinical Observations

**Figure 1:**
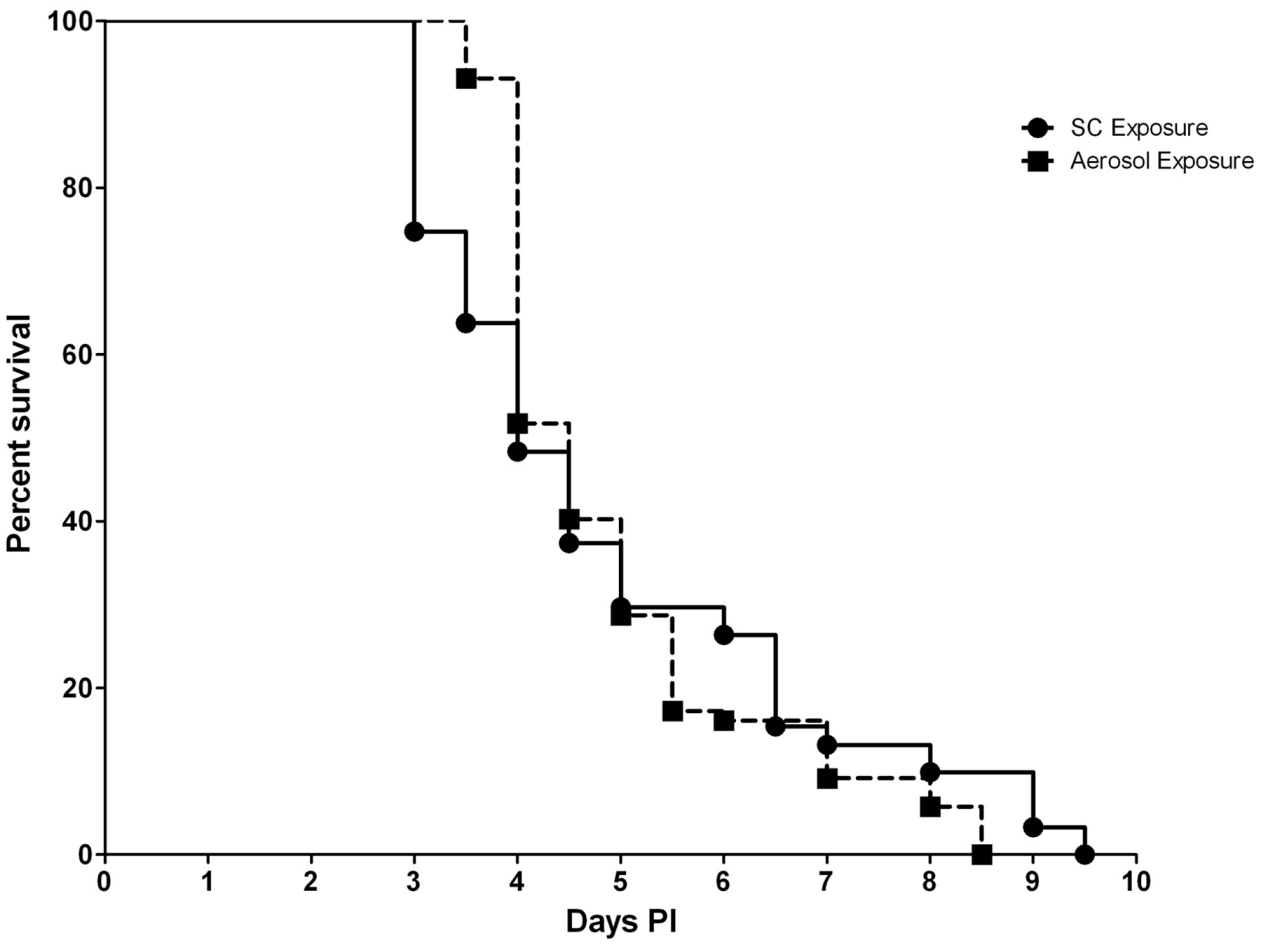
Mouse survival after infection. Percent survival of mice (n = 100) infected SC or by aerosol with 1600 PFU of RVFV.

Exposure to the ZH501 strain of RVFV was uniformly lethal by both SC and aerosol challenge routes. The LD_50_ was determined to be 0.27 PFU and 0.75 PFU for mice exposed SC and by aerosol, respectively (data not shown). When mice were exposed to 1600 PFU, signs of clinical illness become apparent on day 3 PI at which time the mice presented with ruffled fur and a hunched posture. The majority of mice in both groups were euthanized or succumbed to infection between days 3 and 4 PI, with 70% overall mortality from systemic disease by day 5 PI by both routes (Figure 1). The mean time-to-death (MTD) for mice exposed SC was 4.87 days and for mice exposed by aerosol 4.94 days, which was not significantly different.

**Figure 2:**
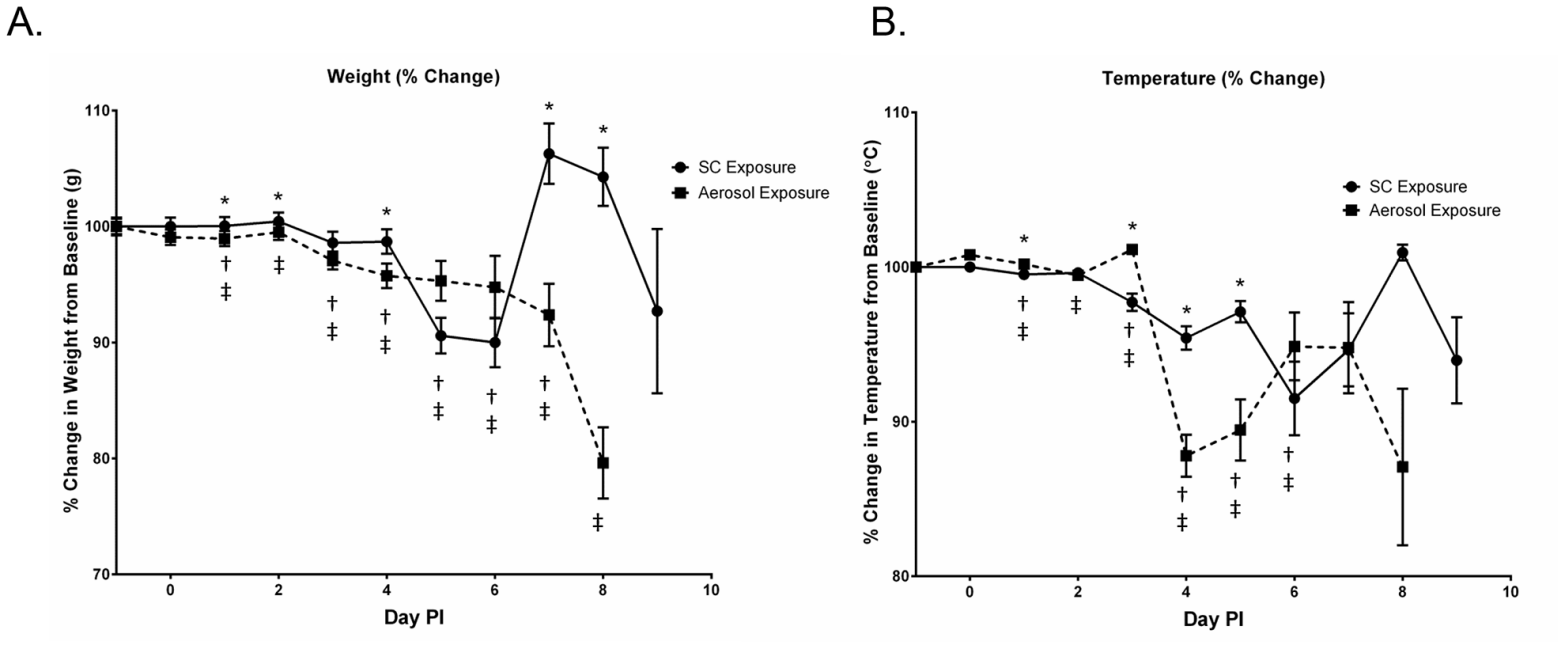
Mouse daily weight and temperature. Percent change in baseline of (A) weights and (B) temperature of mice exposed to RVFV SC or by aerosol (N = 5–100/day PI). The symbols represent the mean value and the error bars represent mean standard deviation. Asterisk (*) indicates significantly different values when comparing SC vs. aerosol exposed mice. Single cross (†) indicates significantly different values for SC exposed mice compared to day −1 uninfected controls. Double cross (‡) indicate significantly different values for aerosol exposed mice compared to day −1 uninfected controls.

Mice exposed by both challenge routes began to lose weight and have changes in their body temperature as early as day 3 PI (Figure 2). Mice exposed by aerosol began to lose significantly more weight (p = 0.0144) and have significantly lower body temperatures (p<0.0001) compared to SC exposed mice on day 4 PI. This trend continued until day 7 PI when mice exposed SC began to gain weight, which was followed by another decrease.

SC exposed mice that succumbed or were euthanized around day 7–9 PI exhibited signs of neurological disease such as hind limb paralysis. Aerosol exposed mice exhibited signs of neurological disease between days 6–8 PI. Therefore, based on disease signs, it appears that the percentage of mice exposed SC and by aerosol that succumbed from systemic disease was 74% and 70% respectively vs. 26% (SC exposure) and 30% (aerosol exposure) that succumbed from neurological disease. For mice exposed by aerosol, 100% mortality from neurological disease was observed 1 day earlier compared to SC exposed mice (day 8.5 PI vs. day 9.5 PI, respectively).

### Clinical Chemistry and Hematology

**Figure 3:**
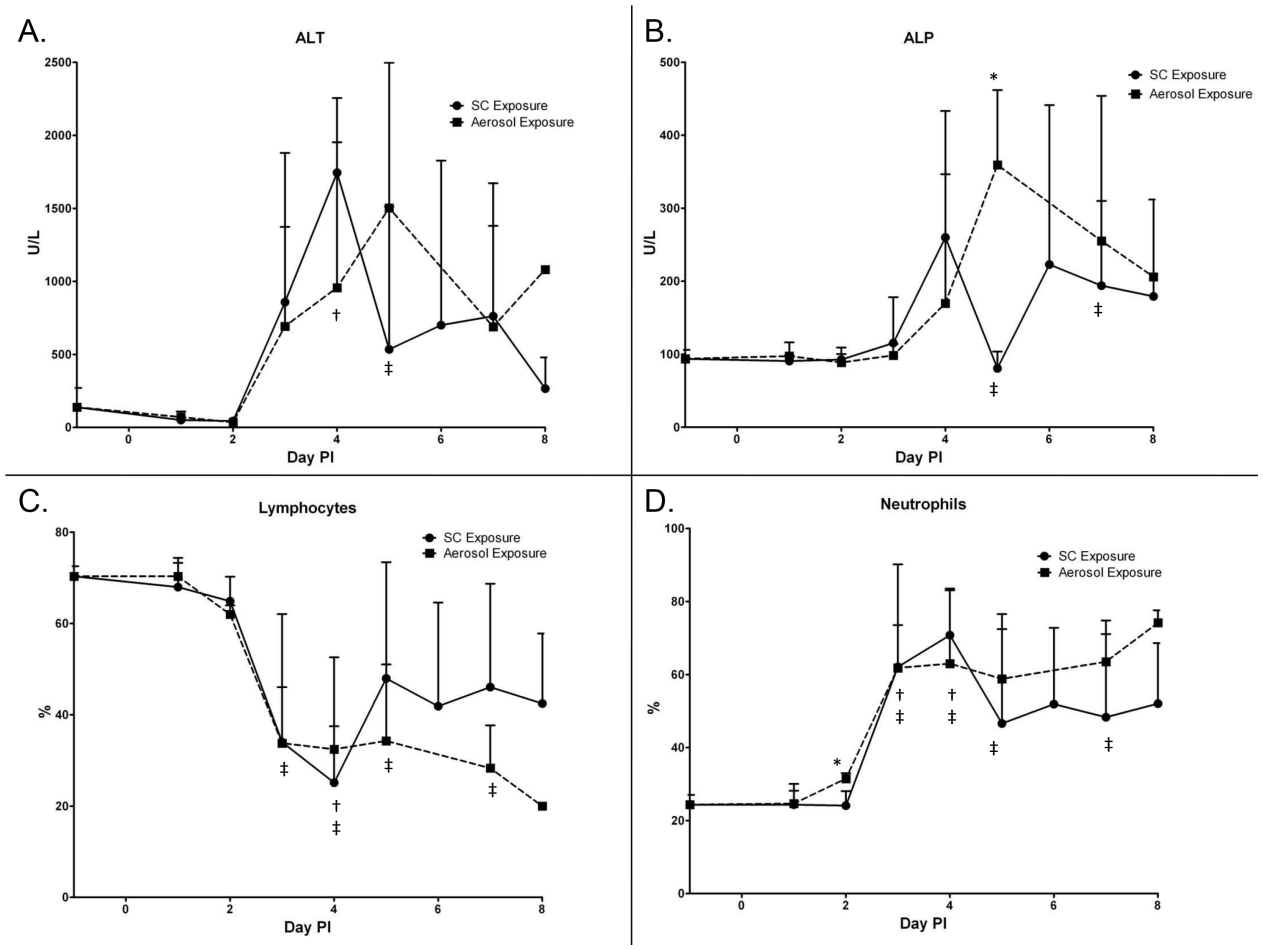
Liver enzyme and hematology results. Analysis of the (A) alanine aminotransferase (ALT), (B) alkaline phosphatase (ALP), (C) percent lymphocytes, and (D) percent neutrophils in RVFV infected mice (N = 4/day PI). The symbols represent the mean concentration and the error bars represent mean standard deviation. Asterisk (*) indicates significantly different values when comparing SC vs. aerosol exposed mice. Single cross (†) indicates significantly different values for SC exposed mice compared to day −1 uninfected controls. Double cross (‡) indicate significantly different values for aerosol exposed mice compared to day −1 uninfected controls.

The blood chemistry and CBC values were determined in the uninfected controls (day 0 PI) and the infected mice on days 1–8 PI (Figure 3). Generally, both aerosol- and SC-infected mice exhibited marked changes in multiple analytes as early as 3 days PI. There were few significant differences in these results between exposure groups, and blood chemistry portrayed a similar picture of the disease courses overall. The liver enzymes ALT (p = 0.0102) and ALP (p = 0.2579) peaked on day 4 PI in SC exposed mice when compared to uninfected controls. In aerosol exposed mice the ALT (p = 0.0198) and ALP (p = 0.0009) levels peaked on day 5 PI (Figure 3A–B), which is one day later compared to SC exposed mice. However, the only statistically significant difference when directly comparing the two exposure routes was higher ALP levels on day 5 PI (p = 0.0102) for mice exposed by aerosol compared to SC exposed mice. A significant amount of variability was observed in mice from both exposure groups. This is most likely due to the study design where mice were randomly sampled and at different stages of RVF disease. Chemistry analyses from mice sampled only when they exhibited signs of end-stage disease were more consistent among animals (data not shown). Other analytes found to be significantly different when comparing mice exposed by aerosol vs. SC included TBIL, CA, PHOS, GLU, and K^+^ on various days PI (data not shown).

CBC results also showed markers of hematological dysfunction that were mostly conserved across both exposure groups. The most drastic was a drop in percent circulating lymphocytes relative to baseline values (p_aero_ = 0.0003, p_sc_ = 0.0541) with a concurrent spike in percent circulating neutrophils (p_aero_ = 0.0004, p_sc_ = 0.0368) 3 days PI in both exposure groups (Figure 3C–D). The most pronounced difference between the aerosol and SC exposure groups was a trend toward recovery of normal values after 3 days PI among SC-infected animals; while the aerosol exposed cohort maintained the same aberrant lymphocytopenia and neutrophilia established 3 days PI through the end of the study. However, the only statistically significant difference (for percent lymphocytes and neutrophils) when directly comparing the results from both exposure groups resulted on day 2 PI where mice exposed by aerosol had slightly higher levels of percent circulating neutrophils (p = 0.0135). This is most likely because significant variability was observed in each cohort of mice, which is similar to the chemistry results. Other hematology values found to be significantly different when comparing mice exposed by aerosol vs. SC included %MO, %BA, RBC, Hb, %HCT, MCV, MCH, MCHC, %RDW, and PLT on various days PI (data not shown).

### Viral Titers

**Figure 4:**
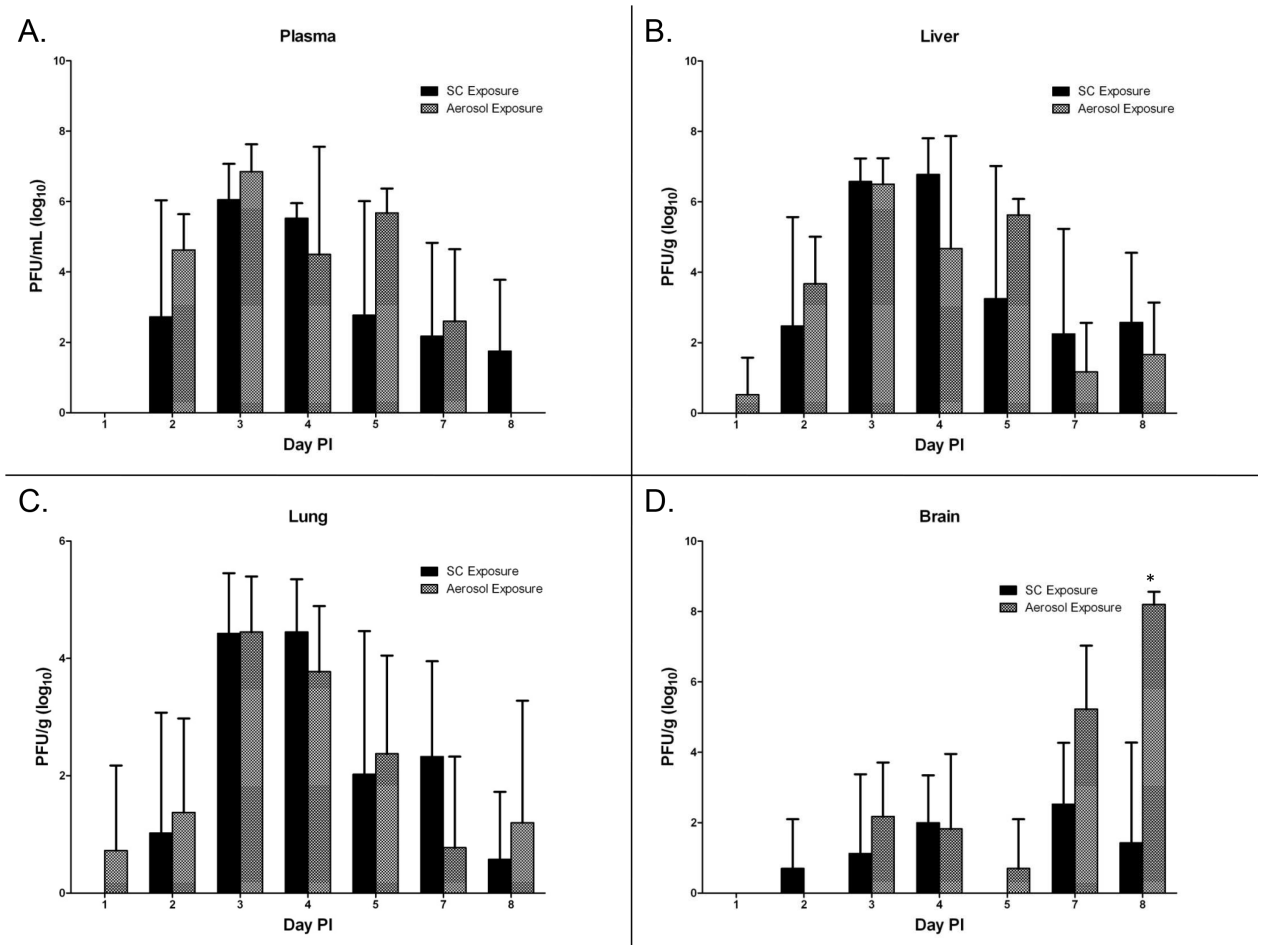
Viral titers. Viral titers in (A) plasma, (B) liver, (C) lung, and (D) brain of RVFV infected mice (N = 4/day PI). Error bars represent mean standard deviation. Asterisk (*) indicates significantly different values when comparing SC vs. aerosol exposed mice.

The viral titers in the plasma, liver, lung, and brain were determined by standard plaque assay on days 1–5 and days 7–8 PI (Figure 4). Both SC and aerosol exposure routes resulted in viremia on day 2 PI, which peaked on day 3 PI for both exposure groups (Figure 4A). Virus was first detected in the liver (Figure 4B) and lung (Figure 4C) from aerosol exposed mice on day 1 PI whereas no virus was detected in the tissues of SC exposed mice on the same day. Virus was detected in all tissues acquired 3 days PI from mice in both exposure groups. Viral titers in the liver and lung were highest 3 days PI in both exposure groups, and then gradually decreased throughout the course of the study in both cases. Viral titers peaked in the brain on day 7 PI for mice exposed SC and on day 8 for mice exposed by aerosol (Figure 4D). Overall, no significant differences resulted in the viral titers in the plasma, liver, and lung from mice exposed either by aerosol or SC. However, mice exposed by aerosol did have significantly higher titers of virus in the brain compared to SC exposed mice on day 8 PI (p<0.001).

### Histopathology

Histological examination of tissues from infected mice exposed by aerosol and SC showed tropism of RVFV for a variety of tissues and cell types throughout the course of infection similar to the results we previously described [14]. Viral antigen was present at various times in epithelial cells (hepatocytes, adrenocortical cells, odontogenic epithelium, olfactory neuroepithelial cells), mesenchymal cells (perineural cells, periosteal and endosteal cells, perivascular cells, bone marrow stromal cells, fibroblastic reticular cells, myocardial cells, vascular smooth muscle cells, adipocytes), neural cells (olfactory neurons and multiple neuronal types in the brain), hematopoietic cells (macrophages and other unidentified cells) and endocrine cells (pancreatic islet cells and pituicytes).

**Figure 5:**
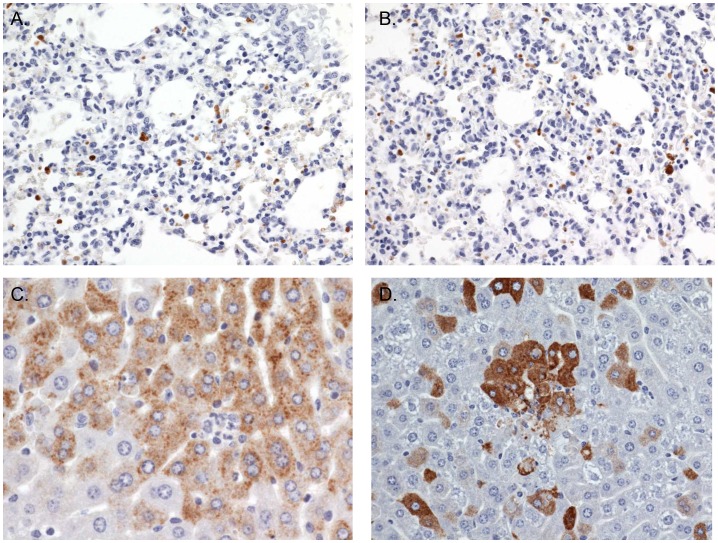
Lung and liver immunohistochemical findings. Immunohistochemical findings in the (A–B) lung and (C–D) liver of RVFV infected mice. Minimal viral antigen is detected in the lungs of (A) SC exposed (20×) and (B) aerosol exposed (20×) mice on day 2 PI. Significant viral antigen was detected on day 4 PI in the liver of mice exposed (C) SC (40×) and (D) by aerosol exposure (40×).

Regardless of the exposure route, only minimal viral antigen was detected in the lungs of mice exposed by aerosol and SC (Figure 5A–B, Table 1). In mice that were exposed by aerosol, scant, weak antigen localization was observed in the lungs of 2 of 4 mice on day 1 PI and in one of 4 mice on day 2 PI. The antigen observed may have been residual antigen deposited in the lungs as respirable sized particles from the aerosol. The mediastinal lymph nodes, which drain the lungs were available for microscopic examination in 2 of 4 mice on day 1 PI, but these regional lymph nodes did not have imunohistochemical evidence of viral infection. In both models, viral antigen localization was observed within intraalveolar cells, alveolar septal cells, and intravascular cells of the lungs. No histological evidence of pneumonia was observed in the lungs of mice exposed by aerosol or SC.

**Table 1:** Assessment of viral antigen in tissues by immunohistochemistry.

		Lung	Liver	Olfactory Epithelium	Olfactory Bulb	Brain
**Aerosol Exposure**	**Day 2 PI**	0–1+	1+	1+	0–1+	0
	**Day 4 PI**	0–1+	3+	2+	0–1+	0
	**Day 7 PI**	0–1+	1+	1+	2+	2+
	**Day 8 PI**	0	1+	2+	3+	4+
**SC Exposure**	**Day 2 PI**	0–1+	1+	0	0	0
	**Day 4 PI**	1+	4+	0	0	0
	**Day 7 PI**	1+	2+	1+	0	0
	**Day 8 PI**	1+	1+	1+	1+	2+

The amount of antigen in a given location was characterized as 0 (none), 1+ (minimal), 2+ (mild), 3+ (moderate), 4+ (extensive). Values represent the average of four mice except in the case where no antigen (0) was noted, which are indicated as a range of the antigen score.

The liver was a clear early and dominant target in mice exposed by aerosol and SC. A few infected hepatocytes were evident as early as day 2 PI (3 of 4 mice for aerosol exposure and 2 of 4 for SC exposure; Table 1). Strong and widespread viral antigen staining of hepatocytes was apparent in livers of both aerosol- and SC-infected mice by day 4 PI (Figure 5C–D, Table 1), which is similar to our previous study results [14]. Also evident on days 3, 4, and 5 PI was damage to hepatocytes ranging from a few to numerous dead cells. Despite extensive infection of, and damage to hepatocytes, clearance of virus from the liver occurred to a great extent in mice that survived to later time points, as reduced amounts of viral antigen were evident by day 8 PI in mice exposed by aerosol and SC.

**Figure 6:**
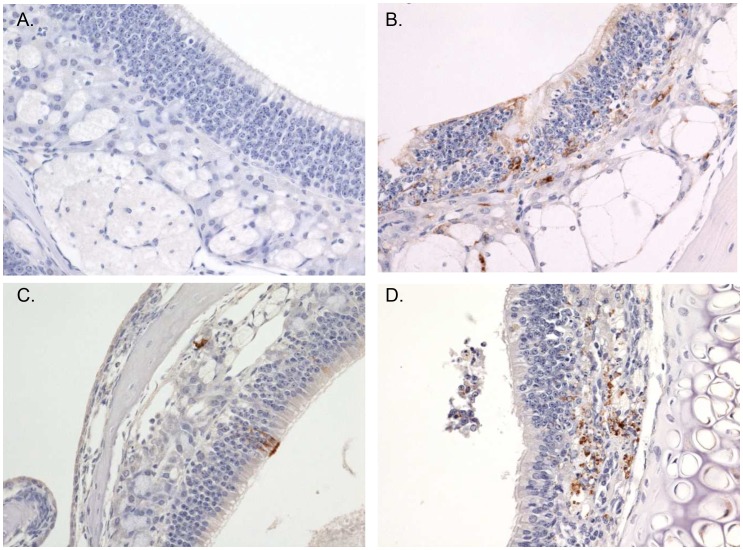
Nasal turbinate immunohistochemical findings. Immunohistochemical findings in the nasal turbinates of mice exposed SC (A, C; left side) or by aerosol exposure (B, D; right side). (A) Immunonegative olfactory neuroepithelium and fila olfactoria on day 4 PI in a SC exposed mouse, 40×. (B) Immunoreactive olfactory neuroepithelium and fila olfactoria on day 4 PI in an aerosol exposed mouse, 40×. (C) Rare, focal immunoreactivity of olfactory neuroepithelium and fila olfactoria on day 7 PI in a SC exposed mouse, 40×. (D) In comparison to SC exposed mice, significantly more viral antigen was detected in the olfactory neuroepithelium and fila olfactoria on day 7 PI in aerosol exposed mice, 40×.

An early target of aerosolized RVFV was the olfactory neuroepithelium lining nasal turbinates, with a few immunolabeled cells present in 4 of 4 mice at day 2 PI and in 3 of 4 mice on day 3 PI (Figure 6, Table 1). Infected cell types included cells morphologically compatible with neuroepithelial cells and cells of the subjacent fila olfactoria. Similar to the liver, the olfactory neuroepithelium exhibited progressive infection in some mice at later time points. In contrast, the same tissues from animals exposed SC were uniformly immunonegative until 7 days PI at which point the nasal turbinates and olfactory neuroepithelium were positive for viral antigen.

**Figure 7:**
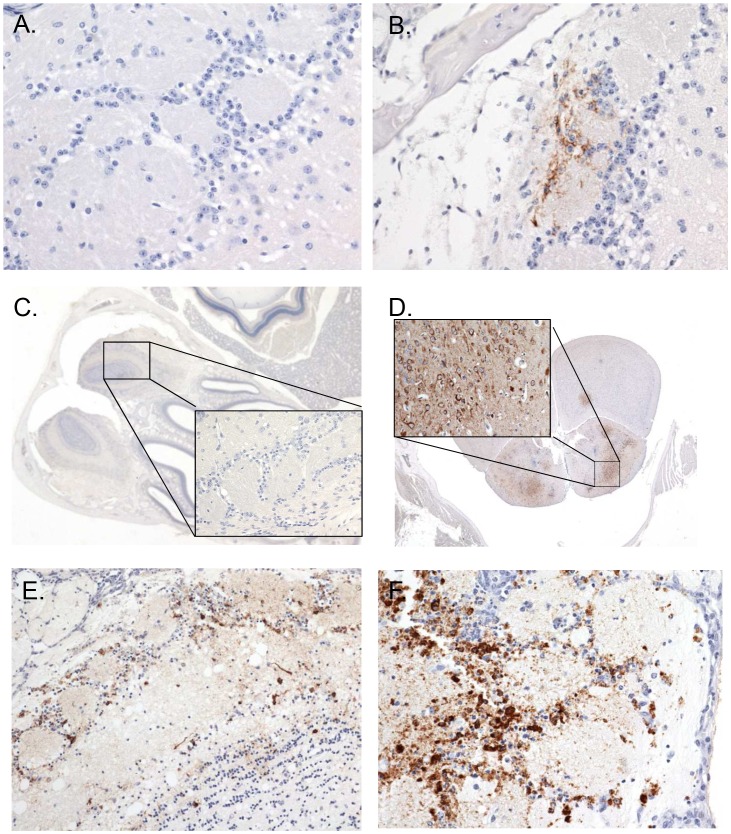
Olfactory bulb immunohistochemical findings. Immunohistochemical findings in the olfactory bulb of mice exposed SC (A, C, E; left side) or by aerosol exposure (B, D, F; right side). (A) Immunonegative cells of the glomerular layer on day 4 PI in a SC exposed mouse, 40×. (B) Immunoreactive cells of the glomerular layer on day 4 PI in an aerosol exposed mouse, 40×. (C) Immunonegative cells of the olfactory bulb (inset depicts the glomerular layer, 40×) on day 7 PI in a SC exposed mouse, 2×. (D) Strongly immunoreactive olfactory neural cells (inset depicts immunoreactive neurons of the glomerular layer, 40×) on day 7 PI in an aerosol exposed mouse, 2×. (E) Moderate amounts of viral antigen in the glomerular layer on day 8 PI in a SC exposed mouse, 40×. (F). Intensely immunoreactive cells in the glomerular layer on day 8 PI in an aerosol exposed mouse, 40×.

**Figure 8:**
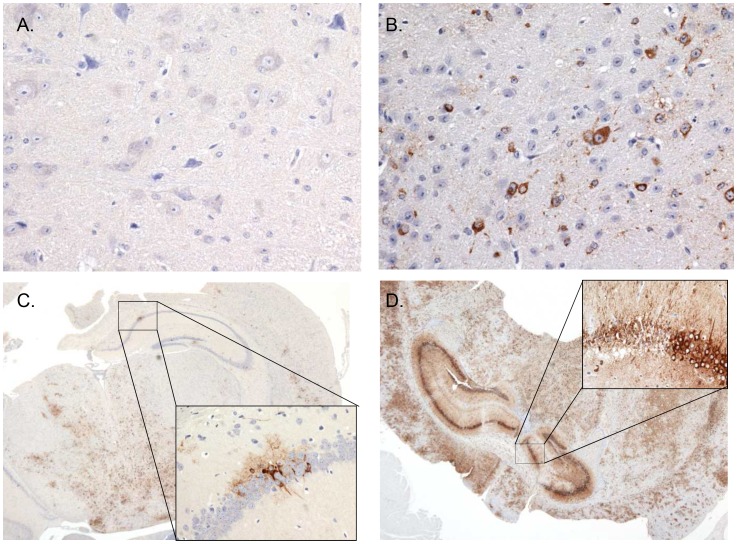
Brain immunohistochemical findings. Immunohistochemical findings in the brain of mice exposed SC (A, C; left side) or by aerosol exposure (B, D; right side). (A) Immunonegative brainstem on day 7 PI in a SC exposed mouse, 40×. (B) Immunoreactive neurons in the brainstem on day 7 PI in an aerosol exposed mouse, 40×. (C) Immunoreactive hippocampus, brainstem, and cerebral areas (inset depicts immunoreactive hippocampal neurons, 40×) on day 8 PI in a SC exposed mouse, 2×. (D) Intensely immunoreactive hippocampus, brainstem, and cerebral areas (inset depicts strongly immunoreactive hippocampal neurons, 40×) on day 8 PI in an aerosol exposed mouse, 2×.

The earliest evidence of neuroinvasion in mice exposed by aerosol was antigen localization limited to the olfactory bulbs, in one mouse on day 2 PI, in one mouse on day 4 PI, and in 3 of 4 mice on day 5 PI. In contrast, viral antigen was not detected in the same region until day 8 PI for mice exposed SC (Figure 7, Table 1). Histological evidence of infection in the brain proper was first apparent in neurons of the brainstem in 1 of 4 mice euthanized on day 5 PI that were exposed by aerosol. For mice exposed SC, histological evidence of infection of the brain occurred on day 6 PI in 1 of 4 mice. Neurons were the main target cell in the brain for both exposure routes. By day 7 PI, 4 of 4 mice had evidence of neuronal infection in the brainstem and other areas of the brain for mice exposed by aerosol. Less viral antigen was detected in the brain of mice exposed SC with 1 of 4 mice exhibiting neuronal infection on day 8 PI (Figure 8, Table 1). Morphologic changes in the brain developed concurrently with the detection of viral antigen in mice exposed by aerosol and SC. The brain lesions were generally characterized as meningoencephalitis with neuronal necrosis.

### Evaluation of Ribavirin against RVFV in the Murine Model

**Figure 9:**
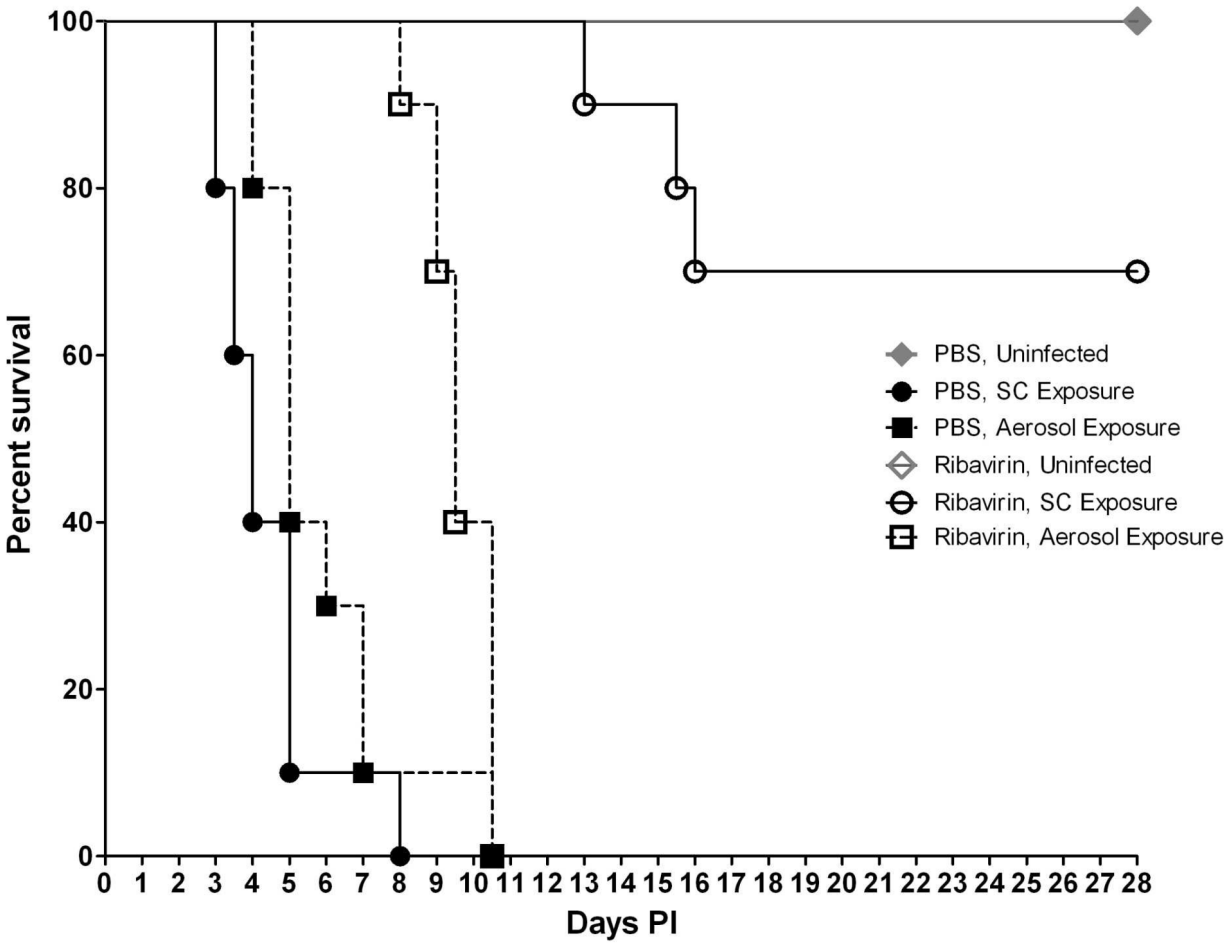
Mouse survival following ribavirin treatment. Percent survival of mice treated with PBS or ribavirin and infected SC or by aerosol with RVFV (n = 10/group).

**Figure 10:**
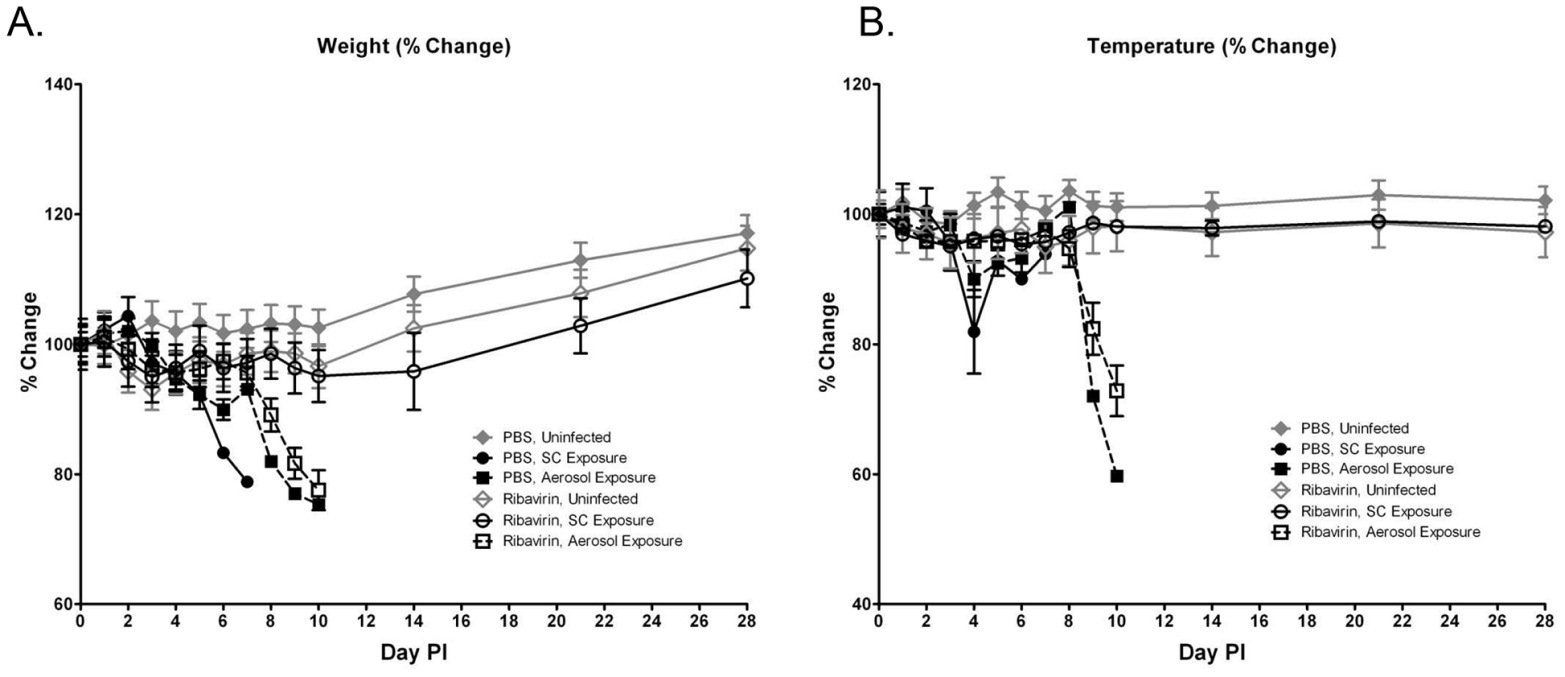
Mouse daily weight and temperature following ribavirin treatment. Percent change in baseline of (A) weights and (B) temperature of mice treated with PBS or ribavirin and exposed to RVFV SC or by aerosol (N = 10/group). Each symbol indicates the mean and the error bars represent mean standard deviation.

Cohorts of mice were treated with PBS as a control or ribavirin prophylactically and exposed to 1951 PFU of RVFV by aerosol or SC. As expected, all uninfected controls survived (Figure 9) and did not exhibit significant decreases in weight or temperature (Figure 10). All mice that were treated with the PBS control and exposed by aerosol or SC were euthanized or succumbed to infection and did have significant decreases in weight and temperature. MTD for PBS injection treatment control aerosol- and SC-exposed groups were 6 and 4.5 days, respectively. Mice that were treated with ribavirin and exposed SC to RVFV showed 70% survival with a MTD of 15 days and did not experience significant changes in weight or temperature. In contrast, ribavirin treated, aerosol-exposed mice showed 0% survival with a MTD of 9.5 days and had significant decreases in weight and temperature (Figure 9 and 10). Non-surviving treated animals exposed SC and by aerosol exhibited signs of neurological disease at the time of death or euthanasia.

**Figure 11:**
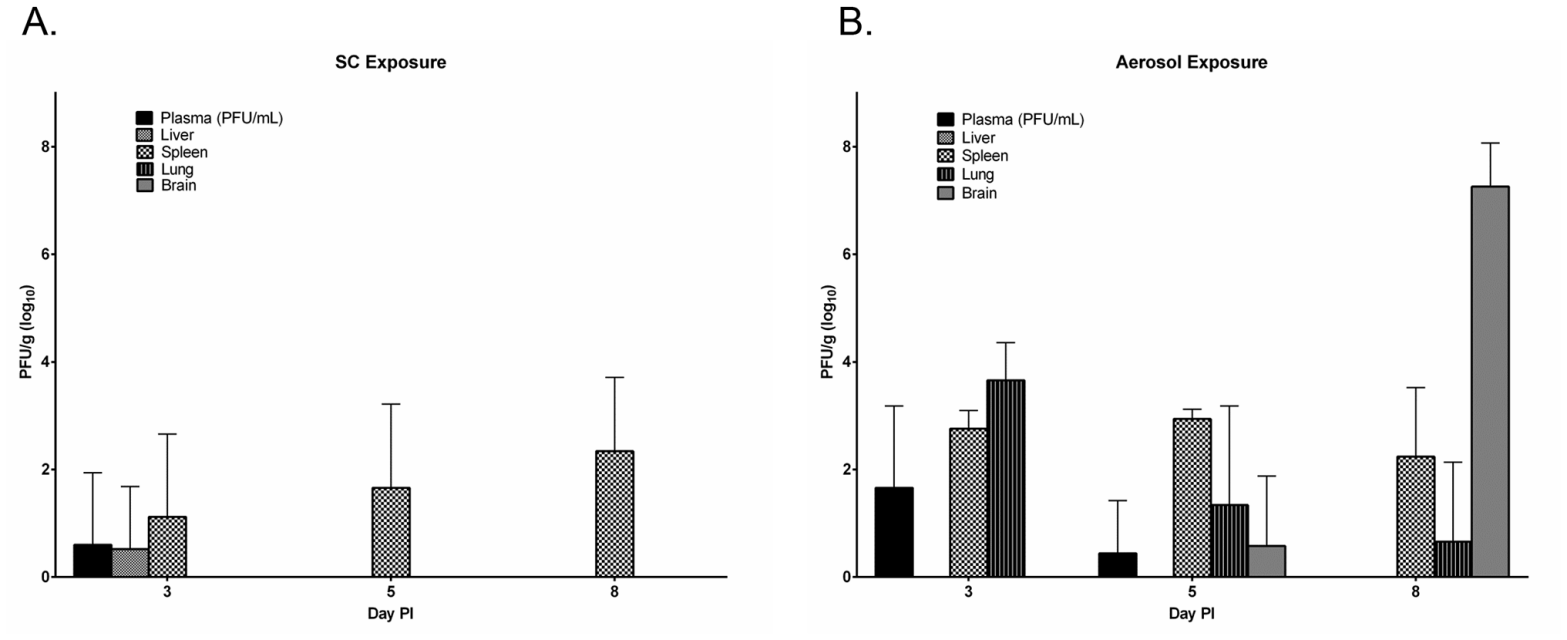
Viral titers in ribavirin treated mice. Viral titers in the plasma and tissues of ribavirin treated mice infected by (A) SC exposure or (B) aerosol exposure (N = 4/day PI). Error bars represent mean standard deviation.

Despite the variances in MTD and survival, there were no significant differences between SC- and aerosol-exposed mice in clinical blood chemistry and hematology values from blood samples acquired 3, 5, and 8 days PI (data not shown). Viral titers from tissues sampled at these same time points demonstrated more virus in tissues from ribavirin treated, aerosol exposed animals compared to SC exposed animals (Figure 11). Virus was only detected in the plasma, liver, and spleen of ribavirin treated, SC exposed mice. When tissues were assessed for viral antigen by IHC, ribavirin was partially effective at reducing the amount of viral antigen in the lungs and liver, but had only a delayed effect on the amount of viral antigen detected in the olfactory epithelium and olfactory bulb of aerosol exposed mice (Table 2). Our histology results also indicate that ribavirin treatment had little or no effect on CNS pathology where ribavirin treated vs. untreated aerosol exposed animals had similar degrees of pathological changes in the olfactory bulb and brain. This is in contrast to ribavirin treated, SC exposed mice, which had no evidence of pathological changes on day 8 PI (Table 3).

**Table 2:** The percentage of mice with viral antigen detected in tissues by immunohistochemistry.

		Lung	Liver	Olfactory Epithelium	Olfactory Bulb	Brain
**Ribavirin Treated, Aerosol Exposure**	**Day 3 PI**	0/5 (0%)	3/5 (60%)	1/5 (20%)	0/5 (0%)	0/5 (0%)
	**Day 5 PI**	1/5 (20%)	0/5 (0%)	4/5 (80%)	3/4 (75%)	0/5 (0%)
**Untreated, Aerosol Exposure**	**Day 3 PI**	2/4 (50%)	4/4 (100%)	3/4 (75%)	0/4 (0%)	0/5 (0%)
	**Day 5 PI**	0/4 (0%)	4/4 (100%)	3/4 (75%)	3/4 (75%)	0/5 (0%)
**Ribavirin Treated, SC Exposure**	**Day 3 PI**	0/5 (0%)	3/5 (60%)	0/5 (0%)	0/5 (0%)	0/5 (0%)
	**Day 5 PI**	NA	NA	NA	NA	NA
**Untreated, SC Exposure**	**Day 3 PI**	1/4 (25%)	4/4 (100%)	0/4 (0%)	0/4 (0%)	0/4 (0%)
	**Day 5 PI**	0/4 (0%)	3/4 (75%)	0/4 (0%)	0/4 (0%)	0/4 (0%)

NA = not available for analysis.

**Table 3:** Evaluation of the CNS pathology in ribavirin treated and untreated mice on day 8 PI.

	Olfactory Bulb	Brain
**Ribavirin Treated, Aerosol Exposure**	++	++
**Untreated, Aerosol Exposure**	++	+++
**Ribavirin Treated, SC Exposure**	−	−
**Untreated, SC Exposure**	+	+

The degree of pathological changes was characterized as − (none), + (minimal to mild), ++ (mild to moderate), and +++ (moderate to marked).

## Discussion

### Pathogenesis of RVFV Following Aerosol or SC Exposure

Because of the possibility of RVFV exposure by multiple routes, it is necessary to discern the differences in pathogenesis and treatment accordingly. In the current study, we first performed detailed comparative pathological analyses on mice infected by aerosol and SC exposure routes in order to determine the pathogenetic events unique to each. Additionally, we evaluated the efficacy of the nucleoside analog ribavirin by examining its effects on survival, weight, temperature, and viral load after either aerosol or SC exposure.

RVF disease in humans and livestock is often characterized by early-onset hepatitis and delayed-onset encephalitis, manifestations which are reproduced in the BALB/c RVFV mouse model exposed SC and by aerosol. Despite the route of exposure, no significant differences in overall survival were observed. For mice exposed by aerosol and SC, the liver was a clear early and dominant target of RVFV, with specific targeting of hepatocytes. Hepatic enzymes in the blood peaked one day earlier (day 4 PI) in SC exposed mice compared to aerosol exposed mice. This small difference may be due to the route of infection of the liver. However, this remains unclear because the primary site of replication for RVFV following SC and aerosol exposure has yet to be determined in the BALB/c mouse model. We speculate that mononuclear phagocytic cells and dendritic cells at the SC injection site would first become infected and migrate to the nearest draining lymph node. The virus would then replicate in the lymph node, resulting in primary viremia and spread to other target organs such as the liver via the bloodstream. This is supported in part by our earlier work that demonstrated viral antigen in mononuclear phagocytic cells and dendritic cells in the lymph nodes from infected mice [14]. Additionally, a study utilizing bioluminescent and fluorescent RVFV in immunodeficient mice demonstrated the importance phagocytic cells as targets for viral infection and following intradermal inoculation of virus, the nearest draining lymph node became the main site of early replication [24].

For mice exposed by aerosol, we observed weak immunohistochemical evidence of viral infection in the lungs, which was not a major target for infection despite the route of exposure. Similar to a previous study of aerosolized RVFV in Swiss Webster mice, pneumonia was not a dominant factor in the pathogenesis of RVFV in mice following aerosol exposure [17]. We speculate that alveolar macrophages might be an important early target cell for infection in the lungs and these cells would then migrate to the mediastinal lymph nodes prior to 1 day PI, which could possibly explain the weak immunohistochemical evidence of viral infection in the lungs. The mediastinal lymph nodes is most likely an important primary site for replication followed by spread to other target organs such as the liver, even though we failed to detect viral antigen by immunohistochemistry. Viral antigen could have been present at a level below the detection level for our assay. Other possible routes for infection of the liver include ingestion of virus with seeding of the liver by the enterohepatic circulation or by vascular spread from the lungs.

Viral titers and immunohistochemistry from brain tissues indicated that mice exposed to RVFV by aerosolization developed neuropathology more rapidly and to a greater effect than SC exposed mice. Early, intense immunohistochemical staining of the nasal turbinates and especially the olfactory bulb among mice in the aerosol exposure group may explain the significant development of neuropathology associated with aerosol exposure. Exposure by aerosolization results in direct exposure of the lung and the nasal turbinate epithelia. Therefore, RVFV viral neuroinvasion likely occurs by viral propagation in nasal turbinate epithelial cells; followed by infection of olfactory nerves, the olfactory bulb, and eventually the higher structures of the brain. This is in contrast to the SC exposure group, in which the less severe evidence of neuropathology and rarity of immunopositive cells in the nasal turbinates and olfactory bulb suggests another mechanism for viral entry into the brain. In a previous study of mice following SC exposure to RVFV, we showed that tissues of the spinal cord and then brainstem become progressively infected prior to onset of fatal pan-encephalitis [15], suggesting that entry to the brain may occur by cephalic progression from the neurons and neuroglia of the spinal cord into the basal structures of the brain. These differences in the neuropathogenesis of RVFV should be taken into account when developing medical countermeasures to protect against peripheral and aerosol exposures. Despite these important differences in the development of neuropathology, no significant difference exists in the overall survival of mice exposed by both exposure routes. This is most likely because mice are highly susceptible to RVFV regardless of the route of exposure. The overall disease outcome is similar with most mice developing acute-onset hepatitis and the remaining mice developing encephalitis. However, with such a highly susceptible model it is important to take into account the progression of disease (not just the disease outcome) when designing and evaluating therapeutics. This key point is highlighted by our evaluation of ribavirin in SC and aerosol exposed mice (discussed below) where we observed significant differences in the outcome.

A recent study by Bales et al. assessed the susceptibility of different inbred rat strains to aerosolized RVFV and determined that Wistar-Furth rats developed a similar disease course and outcome when exposed SC and by aerosol (although not directly compared in the same study), which is similar to our results in the BALB/c mouse model. This is most likely because these two models are highly susceptible to RVFV and it is hard to distinguish differences in survival when high mortality results in a short time frame. In contrast, Bales et al. report that Lewis rats developed fatal encephalitis after aerosol infection, but only mild disease following SC exposure. No pathology data was presented, but Bales et al. hypothesized that aerosolized RVFV may gain access to the central nervous system through the olfactory bulb, which is similar to our results in the mouse model. Interestingly, Lewis rats that survived SC exposure were not protected against subsequent re-challenge by aerosol exposure to the same virus [25]. This observation, along with our findings, implicate the importance for further studies to evaluate the route of neuroinvasion by RVFV, which has important implications for the development of countermeasures to protect against multiple exposure routes.

We observed aberrations in certain hematologic parameters, which were conserved across both exposure route groups. First, early and severe neutrophilia occurred in all mice in both exposure groups, with an 80% average increase in percent circulating neutrophils above baseline values observed 3 days PI. This sharp increase reflects the systemic nature of granulocytic inflammation during acute RVFV infection, and correlates well with histological observation of polymorphonuclear white blood cell infiltration into multiple organ systems; particularly secondary lymphoid and liver tissues 3 days PI. Neutrophilia and polymorphonuclear white blood cell inflammation are often associated with acute tissue damage during infectious diseases; such as viral pneumonia [26], [27] and septic shock [28]. The findings of severe neutrophilia and polymorphonuclear leukocyte tissue inflammation in the present study further implicate the importance of the host innate immune response in RVFV pathogenesis.

### Evaluation of Ribavirin Efficacy against RVFV Following Aerosol or SC Exposures

Ribavirin is a nucleoside analogue and its mechanism of antiviral action is not clear. Previous studies have demonstrated the protective efficacy of ribavirin (administered either prophylactically or post exposure) in mice for preventing the hepatic disease, but some mice were euthanized or succumbed from late-developing encephalitis [18], [20], [21], [29]. Similar to these studies, we observed that ribavirin was partially effective in preventing mortality among mice in the SC exposure group. The ribavirin treated mice lived an average of 10.5 days longer than the untreated control mice that were exposed SC and the mice that were euthanized or succumbed developed late-onset encephalitis. To the best of our knowledge, ribavirin has never been evaluated following an aerosol RVFV exposure. We have demonstrated that ribavirin was ineffective among the aerosol group and all mice succumbed from encephalitis. The ribavirin treated mice that were exposed by aerosol lived an average of 3.5 days longer than the untreated control mice. Our results suggest that ribavirin would be completely ineffective in cases where aerosol exposure occurs. This is most likely because of the limited ability of ribavirin to penetrate the blood-brain barrier. Intranasal or aerosol administration of ribavirin does increase the bioavailability of the drug to the brain [30], [31], which could prove more effective to increase survival.

The pathogenetic differences in RVF disease following exposure by either route offer important insights into the relative efficacy of ribavirin against RVFV infection. In particular, early and aggressive viral invasion of brain tissues following aerosol exposure likely played an important role in ribavirin's failure to prevent mortality among these animals. Tissue titers from animals exposed via aerosol showed early infection and rapid propagation in the brain throughout the study, indicating that virus was able to enter and multiply in cells of the brain unimpeded by ribavirin treatment.

Also of key importance was the lack of infectious virus in the liver of any ribavirin-treated mice in the aerosol exposure group at any time-point, and in the SC group after 3 days PI. This rapid elimination of virus from the liver suggests that early ribavirin treatment is efficacious in preventing the primary hepatic syndrome that correlates with early mortality in the mouse model. Serum viral titers also diminished rapidly and then disappeared in both groups after 5 days PI. This lends further support to the hypothesis that ribavirin only fails to protect mice from RVF viral panencephalitis, which was the cause of morbidity and mortality among mice succumbing to RVF viral challenge in spite of treatment in our current study.

Finally, the significant delay in MTD among mice succumbing to RVFV infection after SC exposure and ribavirin treatment suggests that infection may have resulted in lethal panencephalitis after viral neuroinvasion in nonsurviving mice. As in untreated animals, the spleen appeared to serve as a chronically infected viral reservoir, which may have eventually resulted in viral seeding of the basal brain structures via the spinal cord as previously described [14]. While we failed to recover infectious virus from brain tissue in the SC exposure group days 3, 5, or 8 PI; it is likely that direct infection of the brain occurred closer to the MTD, which was on 15 days PI. Viral titers in tissues of treated mice suggest the importance of early viral neuroinvasion in aerosol-exposed mice as a contributor to mortality. Despite a failure to detect infectious virus in plasma at the same time-point, brain tissues collected 8 days PI yielded average viral titers of approximately 8 log_10_ PFU/g. This is equivalent to the titer observed in brain tissue from untreated, terminally sampled animals; and therefore indicates that RVFV proliferation in the brain may be unaffected by ribavirin treatment.

Despite the proven activity of ribavirin against RVFV in rodent and non-human primate models [20], it has not been thoroughly evaluated for the treatment of RVFV infection in humans. During the Saudi Arabia outbreak in 2000, a small-scale, randomized, placebo-controlled clinical trial evaluating ribavirin to treat severe RVFV infections was conducted [32]. Although the study was inconclusive, results suggested that an increased incidence of the encephalitic form of the disease was observed in cases treated with ribavirin (P. Rollin, presented at the Treatment of Viral Hemorrhagic Fever Workshop, Bethesda, MD, 24–27 February 2007). This study and our results highlight the need for more candidate antivirals to treat RVFV infection, especially in the case of a potential aerosol exposure.

In conclusion, our results indicate the utility of the mouse model of RVFV infection as a test system for antiviral therapeutics efficacy. They also highlight the differences in pathogenesis following exposure to either aerosolized or SC-injected RVFV; as well as the effect of exposure route on outcome of disease. Therefore, future evaluation of vaccines and antivirals should be evaluated following multiple exposure routes.
